# Comprehensive analysis of the pseudogenes of glycolytic enzymes in vertebrates: the anomalously high number of GAPDH pseudogenes highlights a recent burst of retrotrans-positional activity

**DOI:** 10.1186/1471-2164-10-480

**Published:** 2009-10-16

**Authors:** Yuen-Jong Liu, Deyou Zheng, Suganthi Balasubramanian, Nicholas Carriero, Ekta Khurana, Rebecca Robilotto, Mark B Gerstein

**Affiliations:** 1 Department of Surgery, Beth Israel Deaconess Medical Center, Harvard Medical School, 110 Francis Street, Boston, MA, USA; 2 Department of Molecular Biophysics and Biochemistry, P.O. Box 208114, Yale University, New Haven, CT 06520, USA; 3 Albert Einstein College of Medicine of Yeshiva University, Department of Neurology, Rose F. Kennedy Center, 1410 Pelham Parkway South, Room 915B, Bronx, NY 10461, USA; 4 Program in Computational Biology and Bioinformatics, Yale University, New Haven, CT 06520, USA; 5 Department of Computer Science, Yale University, Bass 432, 266 Whitney Avenue, New Haven, CT 06520, USA

## Abstract

**Background:**

Pseudogenes provide a record of the molecular evolution of genes. As glycolysis is such a highly conserved and fundamental metabolic pathway, the pseudogenes of glycolytic enzymes comprise a standardized genomic measuring stick and an ideal platform for studying molecular evolution. One of the glycolytic enzymes, glyceraldehyde-3-phosphate dehydrogenase (GAPDH), has already been noted to have one of the largest numbers of associated pseudogenes, among all proteins.

**Results:**

We assembled the first comprehensive catalog of the processed and duplicated pseudogenes of glycolytic enzymes in many vertebrate model-organism genomes, including human, chimpanzee, mouse, rat, chicken, zebrafish, pufferfish, fruitfly, and worm (available at ). We found that glycolytic pseudogenes are predominantly processed, i.e. retrotransposed from the mRNA of their parent genes. Although each glycolytic enzyme plays a unique role, GAPDH has by far the most pseudogenes, perhaps reflecting its large number of non-glycolytic functions or its possession of a particularly retrotranspositionally active sub-sequence. Furthermore, the number of GAPDH pseudogenes varies significantly among the genomes we studied: none in zebrafish, pufferfish, fruitfly, and worm, 1 in chicken, 50 in chimpanzee, 62 in human, 331 in mouse, and 364 in rat. Next, we developed a simple method of identifying conserved syntenic blocks (consistently applicable to the wide range of organisms in the study) by using orthologous genes as anchors delimiting a conserved block between a pair of genomes. This approach showed that few glycolytic pseudogenes are shared between primate and rodent lineages. Finally, by estimating pseudogene ages using Kimura's two-parameter model of nucleotide substitution, we found evidence for bursts of retrotranspositional activity approximately 42, 36, and 26 million years ago in the human, mouse, and rat lineages, respectively.

**Conclusion:**

Overall, we performed a consistent analysis of one group of pseudogenes across multiple genomes, finding evidence that most of them were created within the last 50 million years, subsequent to the divergence of rodent and primate lineages.

## Background

Pseudogenes are inheritable genomic sequences sharing large amounts of sequence similarity to genes but exhibit limited or altered functionality because of disablements. They occur in many prokaryotic and eukaryotic genomes [1-11], but the abundance of pseudogenes is specific to each species. Pseudogenes comprise a significant portion of mammalian genomes and can be found primarily in non-coding regions such as intergenic regions and introns. Because of the high level of sequence similarity shared with the parent genes, the genes from which they were mostly likely generated, it has been a difficult task to biochemically and computationally distinguish pseudogenes from genes. Resolving the functional differences between genes and pseudogenes in spite of their sequence similarity would increase our understanding of regulatory mechanisms that determine gene expression [12,13].

Pseudogenes can be classified into two main types, processed and duplicated [6]. Processed pseudogenes are generated via retrotransposition of the mRNA of their parent genes. After mRNAs of the parent genes are transcribed in the usual fashion by RNA polymerases, they are reverse transcribed and integrated into genomic DNA by reverse transcriptases and endonucleases encoded by long interspersed nuclear elements (LINEs) in primates and humans [14,5,17]. Because these pseudogenes are generated through mRNA intermediates, they are notable for their lack of introns, spliced out during mRNA maturation. On the other hand, duplicated pseudogenes are generated via direct DNA-to-DNA duplication followed by integration into genomic DNA and eventual disablement [18]. They retain most of the exon-intron arrangements with possible duplication of upstream and downstream regions.

We have developed computational methods for cataloguing processed and duplicated pseudogenes [19,3,4,20,2]. First we identify pseudogene candidates by aligning the genome in all six frames of the translated amino acid sequences to the known proteins in the organism [21]. Then we distinguish pseudogenes from their parent genes by identifying disablements such as insertions, deletions, and nonsense mutations, as these would interfere with the potential transcription and translation of the pseudogenes into a fully functional protein.

Because pseudogenes are released from the pressures of natural selection, they capture the sequences of genes at points in time and are subsequently subject to mutations at a neutral rate [22]. Understanding the subtleties of pseudogenes that effect their inactivation would aid in predicting genes *de novo *from genome sequences [23-25]. In addition to their passive role as genetic fossils, the functional roles of pseudogenes are still being characterized. Pseudogenes have been found to interact with the mRNA of their parent gene [26-28]. Some pseudogenes have also been implicated in chromosomal recombination and gene conversion events leading to diseases because of high sequence homology to their parent genes [7,29]. Others have been reactivated and become fully expressed variants of their parent genes [30].

In order to characterize the factors influencing the generation of pseudogenes, it is useful to study a selected set of genes that are common to multiple species and have many associated pseudogenes [22]. We identified such a set that encodes the enzymes in glycolysis, a fundamental metabolic pathway conserved since ancient anaerobic prokaryotes. Using our pseudogene pipeline, we assembled the first detailed catalog of the processed and duplicated pseudogenes of glycolytic enzymes in the well-annotated eukaryotic genomes: human, chimpanzee, mouse, rat, chicken, zebrafish, pufferfish, fruitfly, and worm genomes [20,31-39]. By comparing pseudogenes of orthologous genes in multiple genomes, we are able to identify general characteristics as well as species-specific characteristics. The dates of species divergence can be used as landmarks in the temporal evolution of the glycolytic pseudogenes.

From this analysis, we found that the number of processed and duplicated pseudogenes of GAPDH, as well as its spermatogenic isozyme, far exceeded the numbers of other glycolytic pseudogenes, and for this reason, most of the present work focuses on GAPDH specifically. In order to look for an evolutionary explanation for the large number of GAPDH pseudogenes, we matched orthologous regions by extensive synteny analysis, using genomes that had sufficiently complete and intact annotations and significant numbers of GAPDH pseudogenes, namely the human, mouse, and rat genomes. After considering various methods that aligned large genomic segments by nucleotide sequences [40], we decided to align the genomes using orthologous genes as anchors. Then, after applying Kimura's two-parameter model for neutral evolution [41], we calculated a burst in retrotranspositional activity dating to about 26 million years ago. This relative recentness is consistent with the low numbers of GAPDH pseudgenes syntenic between the primate and rodent lineages. Our study documents a careful analysis of a group of pseudogenes in multiple organisms, contrasting against recent studies devoted to draft pseudogene annotation of individual genomes and attempting to date the burst in retrotransposition [28,42].

## Methods

### Genomic sequences and annotated genes

The human (*Homo sapiens*) NCBI 35 assembly, the chimpanzee (*Pan troglodytes*) 4× shotgun assembly released on November 13th 2003 from the Chimpanzee Sequencing Consortium, the mouse (*Mus musculus*) NCBI m34 assembly, the rat (*Rattus norvegicus*) assembly version 3.4 November 2004 update from the Rat Genome Project, and the chicken (*Gallus gallus*) first draft assembly were downloaded from ENSEMBL release 33. The zebrafish (*Danio rerio*) assembly version 7 (Zv7) released on 13 July 2007, the pufferfish (*Tetraodon nigroviridis*) assembly version 7, the fruitfly (*Drosophila melanogaster*) BDGP assembly release 5, and worm (*Caenorhabditis elegans*) WormBase 180 frozen database were downloaded from ENSEMBL release 49. Gene annotations, their intron and exon positions, and their protein sequences were also obtained from ENSEMBL. The segmental duplications for the human NCBI 35 assembly were obtained from .

Computer programs were written in Perl and GNU Bash to collect and process data. The Perl API provided by ENSEMBL was used to query releases 33, 36, and 49 of its genome databases.

### Pseudogene pipeline

We used a pseudogene pipeline containing separate routines to identify processed and duplicated pseudogenes. The pipeline had been tested on large parts of the human genome [3,4,28,20,43]. On one hand, protein sequences were used to query each genome for processed pseudogenes. Minimal thresholds for identifying processed pseudogenes were optimized at 40% sequence identity and 70% alignment without an insertion longer than 60 nucleotides. Pseudogene candidates that did not meet the second criterion were considered pseudogene fragments. On the other hand, nucleotide sequences spanning a parent gene's exons with 50-nucleotide extensions in both 5' and 3' directions were used to query each genome for duplicated pseudogenes. Repetitive sequences and exons were masked in all candidate matches for processed and duplicated pseudgenes. Please see the methods section of Zheng and Gerstein (2006) for thorough specifications of the pseudogene pipeline [43].

To examine the sensitivity of the pseudogene pipeline, we varied both the percent identity and e-value threshold used for the identification of the pseudogenes in the mouse genome. The total number of pseudogenes varied from 16,963 to 15,884 while the degree of similarity to the parent protein was incremented from 25% to 50%, which constituted a dramatic range. This showed that the number of pseudogenes did not change significantly with the sequence identity parameter, about 40 pseudogenes per 1% increase in sequence similarity. We used an identity threshold of 40%, which yielded 16,730 pseudogenes. We performed similar sensitivity analyses for other parameters and present those results in Additional File 1.

### Synteny

**Figure 1:**
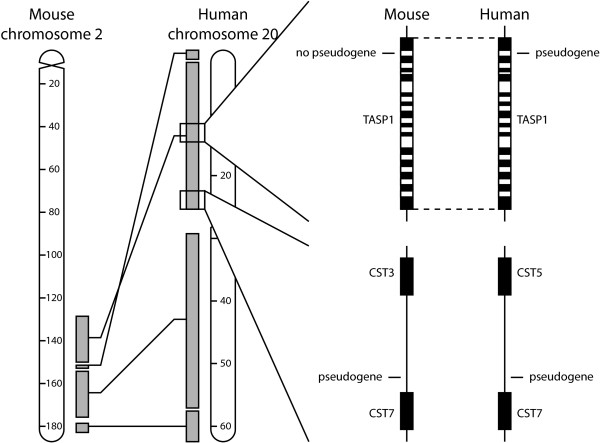
**Syntenic analysis**. Syntenic alignment using orthologous genes as anchors. In the example at top right, a orthologous pair of TASP1 genes is used as an anchor to determine that there is no syntenic mouse pseudogene corresponding to a human GAPDH pseudogene located in an intron of TASP1. In the example at bottom right, two orthologous pairs of CST genes are used as anchors to identify a syntenic pair of intergenic regions, in which we found a syntenic pair GAPDH pseudogenes. Solid and open bars indicate exons and introns, respectively.

Syntenic analysis was conducted between two genomes using orthologous genes as anchors (Figure 1). A pair of GAPDH pseudogenes found in two genomes was considered a syntenic pair if it was flanked by the same two anchors. Gene orthology was assigned according to the annotations in ENSEMBL release 33. The human, mouse, and rat genomes were used for this analysis because they offered the most complete genomic annotations. We considered including the chimpanzee genome, but with its draft status and because it had only recently diverged from the human genome 5.4 million years ago, the chimpanzee genome would not have contributed significantly to the analysis. In contrast, the mouse-rat divergence occurred 41 million years ago and the human-murine divergence occurred 91 million years ago [44].

### Pseudogene ages

At the nucleotide level, we aligned pairs of orthologous GAPDH genes to each other and pairs of syntentic GAPDH pseudogenes to each other [45-47]. As shown in Table 1, nucleotide differences (*P *= fraction of transitions and *Q *= fraction of transversions) were used to calibrate Kimura's two-parameter model with the assumption that they began to accumulate *T *million years ago at the times of species divergence [41]. The divergence times between each species pair were 91 million years ago for the human-mouse divergence, 91 million years ago for the human-rat divergence, and 41 million years ago for the mouse-rat divergence [44]. The rates of transition and transversion mutations, *α *and *β*, respectively, were calculated by Equations 8-9 in Kimura (1980) as follows.

**Table 1:** Nucleotide differences

	**Transitions**	**Transversions**	**Total Nucleotides Aligned**
human ⇔ mouse	508	399	2509
human ⇔ rat	369	269	1710
mouse ⇔ rat	11046	6307	102905

From our human-mouse, human-rat, and mouse-rat synteny analysis, each pair of syntenic GAPDH pseudogenes were aligned and their nucleotide differences were totaled in each pairwise genome comparison.



The parameters {(*α*_*i*_, *β*_*i*_)|*i *∈ {human-mouse, human-rat, mouse-rat}} were calculated for GAPDH genes and pseudogenes for each pairwise comparison among human, mouse, and rat. We solved for the species-specific rates of transitions as follows.



**Figure 2:**
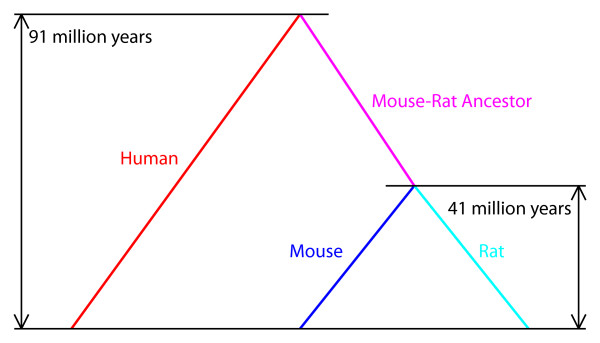
**Human-mouse-rat divergences**. Rates of transitions and transversions were calculated for the human, mouse, and rat genomes as well as the presumed mouse-rat ancestral genome, in order to compensate for the 50 million years between the human-murine divergence and mouse-rat divergence.

The same equations are used, substituting *β*'s for *α*'s, to solve for species-specific rates of transversions. *α*_mouse-rat-ancestor _and *β*_mouse-rat-ancestor _were also calculated for the common ancestor of mouse and rat, in order to account for the time lapse of 50 million years between the human-murine divergence and mouse-rat divergence (Figure 2). The resultant values of *α*_human_, *β*_human_, *α*_mouse_, *β*_mouse_, *α*_rat_, *β*_rat_, *α*_mouse-rat-ancestor_, and *β*_mouse-rat-ancestor _are shown in Table 2. These parameters were then used to calculate the age of each GAPDH pseudogene from the nucleotide differences between it and its parent gene in the same species by solving for *T *in Equation 10 in Kimura (1980) as follows

**Table 2:** Kimura model parameters

		**Human**	**Mouse**	**Rat**	**Mouse-Rat Ancestor**
Genes	*α*	7.15 × 10^-4^	4.90 × 10^-4^	7.28 × 10^-4^	8.02 × 10^-4^
	*β*	2.75 × 10^-4^	1.17 × 10^-4^	1.81 × 10^-4^	3.77 × 10^-4^

Pseudogenes	*α*	1.84 × 10^-3^	1.20 × 10^-3^	1.94 × 10^-3^	2.06 × 10^-3^
	*β*	5.22 × 10^-4^	4.14 × 10^-4^	3.83 × 10^-4^	6.24 × 10^-4^

Rates of transition and transversion substitutions per million years, represented by *α *and *β*, respectively, in Kimura's two-parameter model of nucleotide substitution.



where *α *is taken to be the averaged transition rate for genes and pseudogenes and *β *is taken to be the averaged transversion rate for genes and pseudogenes.

For mouse and rat pseudogenes older than 41 million years, *α *and *β *in the previous equation are replaced with



and



in order to accomodate the nucelotide substitution rates in the common ancestor of mouse and rat.

In these calculations, we derive different rates of nucleotide substitution in genes and pseudogenes because genes are subject to pressures of natural selection whereas pseudogenes are not. Although Kimura's model assumes neutral rates of nucleotide substitutions, we use it as an approximation of the mutation rates of the GAPDH genes for the sake of consistency, perhaps yielding conservative estimates or upper bounds on the ages of pseudogenes.

## Results

### Pseudogene abundances

We assembled a comprehensive catalogue of the processed and duplicated pseudogenes of genes encoding glycolytic enzymes in the human, chimpanzee, mouse, rat, chicken, zebrafish, pufferfish, fruitfly, and worm genomes (Table 3, ). The chicken, zebrafish, pufferfish, fruitfly, and worm genomes contain the least number of GAPDH pseudogenes, none or almost none for each enzyme. The human and chimpanzee genomes both contain moderate numbers. The mouse and rat genomes contain the most, approximately five times as many as the primate genomes. The relative abundances for both processed and duplicated pseudogenes among the vertebrate genomes shows a consistent trend for each glycolytic enzyme: chicken/zebrafish/pufferfish/fruitfly/worm << primates << rodents. However, as previously reported, GAPDH surpasses the other glycolytic enzymes by far in pseudogene abundance (*p *= 0.0023 by Kolmogorov-Smirnov test), followed at a distant second by LDH. Processed pseudogenes outnumber duplicated pseudogenes in all the genomes except chicken, zebrafish, pufferfish, fruitfly, and worm.

**Table 3:** Processed/duplicated pseudogenes

	**Human**	**Chimp**	**Mouse**	**Rat**	**Chicken**	**Zebrafish**	**Pufferfish**	**Fruitfly**	**Worm**
HK	1/0 [4]	1/2	0/1 [3]	- [3]	0/2	-	-	-	-
GPI	- [1]	-	1/0 [1]	- [1]	-	-	-	-	-
PFK	- [3]	-	- [3]	- [3]	-	0/1	-	-	-
ALDO	1/1 [3]	1/1	11/0 [2]	7/0 [3]	0/1	-	-	-	-
TPI	3/0 [1]	2/1	6/1 [1]	3/1 [1]	-	-	-	-	-
GAPDH	60/2 [2]	47/3	285/46 [2]	329/35 [2]	0/1	-	-	-	-
PGK	1/1 [2]	1/2	2/0 [2]	12/0 [1]	-	-	-	-	-
PGM	12/0 [2]	13/1	9/0 [2]	3/0 [2]	-	-	-	-	-
ENO	1/0 [3]	1/2	12/1 [3]	36/3 [3]	-	-	-	-	-
PK	2/0 [2]	3/0	10/3 [2]	4/1 [1]	-	-	-	-	-
LDH	10/2 [3]	9/1	27/7 [3]	25/4 [3]	-	-	-	-	-

**Total**	**97**	**91**	**422**	**463**	**4**	**1**	**0**	**0**	**0**

Numbers of pseudgenes (processed/duplicated) and [known parent gene isozymes] for each glycolytic enzyme. The numbers of known parent gene isozymes are shown only for human, mouse, and rat because they constitute the most completely annotated genomes in ENSEMBL. A dash '-' indicates that there are no processed or duplicated pseudogenes derived from a particular enzyme in a particular vertebrate genome. The chicken, zebrafish, pufferfish, fruitfly, and worm genomes contain very few or no pseudogenes, the human and chimpanzee genomes a moderate number, and the mouse and rat genomes significantly more. The pseudogene copy number for GAPDH far outnumbers those of any other glycolytic enzyme. Abbreviations: HK, hexokinase; GPI, glucose-6-phosphate isomerase; PFK, 6-phosphofructokinase; ALDO, aldolase; TPI, triose-phosphate isomerase; GAPDH, glyceraldehyde-3-phosphate dehydrogenase; PGK, phosphoglycerate kinase; PGM, phosphoglycerate mutase; ENO, enolase; PK, pyruvate kinase; LDH, lactate dehydrogenase.

### Overall distribution

**Figure 3:**
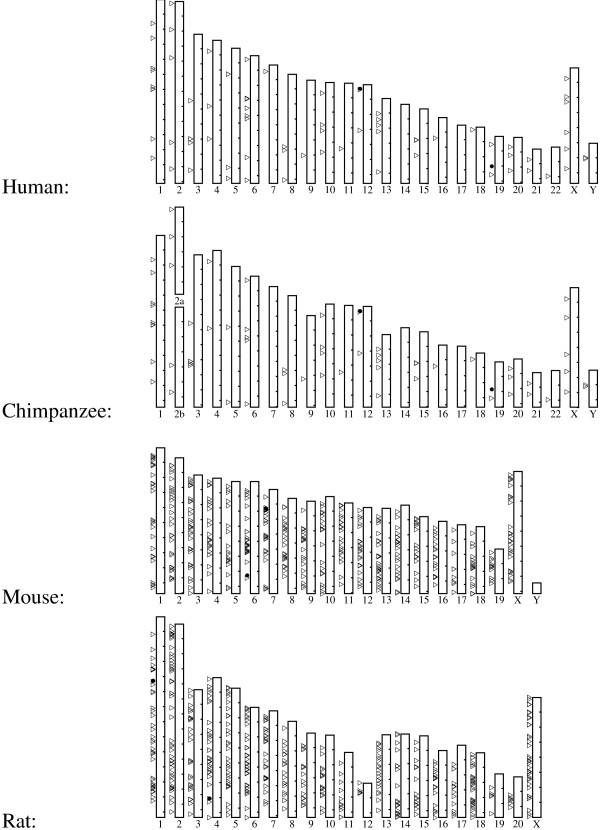
**Pseudogene locations**. Chromosomal distribution of GAPDH pseudogenes in the human genome. Pseudogenes are marked by triangles. The parent genes are marked by solid circles, occuring on human chromosomes 12 and 19 (spermatogenic), chimpanzee chromosomes 12 and 19 (spermatogenic), mouse chromosomes 6 and 7 (spermatogenic), and rat chromosome 1 (spermatogenic) and 4. The tick marks along the right edge of a chromosome mark 20 million base pair intervals, starting from the top.

We mapped the chromosomal locations of the GAPDH pseudogenes in each genome. Figure 3 shows that GAPDH pseudogenes are distributed throughout the human, chimpanzee, mouse, and rat genomes, occuring on all or almost all chromosomes. While clusters of pseudogenes occur at some locations, the overall distribution appears to be uniform and shows no bias towards or against the locations of the parent genes. The other genomes we studied are not shown here because of their scarcity of processed and duplicated pseudogenes.

### Evolutionary analysis with synteny and mutation

**Figure 4:**
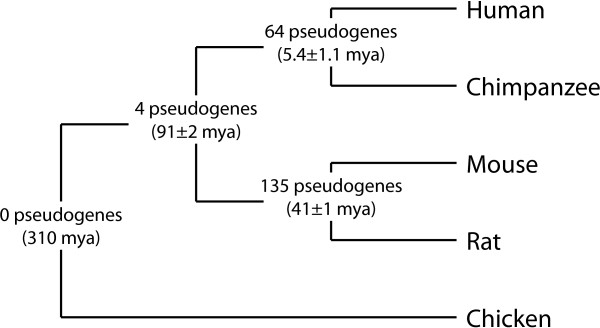
**Phylogeny and numbers of syntenic pseudogenes**. Phylogenic tree relating human, chimpanzee, mouse, rat, and chicken. Branch points are labeled with the number of syntenic GAPDH pseudogenes between the two branches and the approximate date of divergence. Branch lengths are not drawn in proportion to elapsed time.

To investigate the evolution of GAPDH pseudogenes, we attempted to identify syntenic relationships among them. As demonstrated by Figure 1, orthologous genes were used as anchors to delimit regions syntenic between two genomes. Table 4 shows the number of syntenic pseudogenes in each species pair. There were many pairs of pseudogenes syntenic between human and chimpanzee and between mouse and rat while there were very few pairs syntenic between the primate and rodent genomes, suggesting either recent pseudogene production occurring after the primate-rodent divergence or degradation beyond recognizability of pseudogenes older than 75-100 million years (Figure 4).

**Table 4:** Number of syntenic pseudogene pairs

	**Number of Syntenic Pseudogene Pairs**
human ⇔ chimp	64
human ⇔ mouse	4
human ⇔ rat	3
mouse ⇔ rat	135

Number of pseudogene pairs found to be syntenic between two species. The more closely related the pair of species, the greater the number of syntenic pseudogenes, as illustrated by the human-chimp and mouse-rat comparisons.

**Figure 5:**
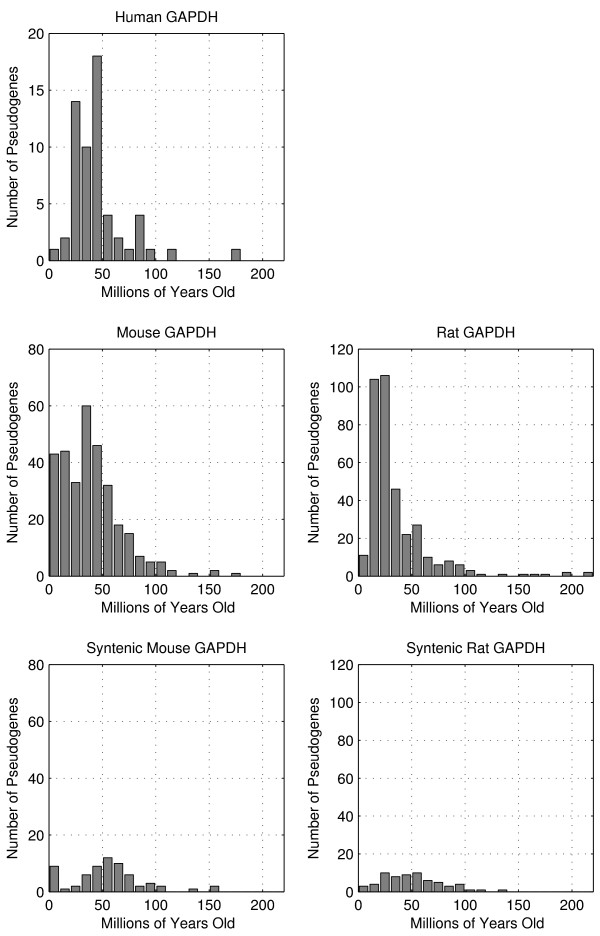
**Pseudogene ages**. Top three panels: Distributions of GAPDH pseudogenes by age in the human, mouse, and rat genomes. There appear to be three distinct bursts in retrotransposition which gave rise to GAPDH pseudogenes centered around medians (middle 50%) of 42.0 million years ago (26.4-49.3 million years) in human, 36.3 million years ago (17.4-52.8 million years) in mouse, and 25.9 million years ago (17.6-40.9 million years) in rat. Pairwise Kolmogorov-Smirnov testing shows that the age distributions among these three genomes are statistically different, with p-values of 0.01 (human-mouse), 7 × 10^-7 ^(human-rat), and 7 × 10^-10 ^(mouse-rat). Bottom two panels: Distributions of GAPDH pseudogenes syntenic between mouse and rat. Although the majority did occur before the mouse-rat divergence 41 million years ago, there is some noise or variation in nucleotide substitutions.

We applied Kimura's two-parameter model of nucleotide substitution to the orthologous GAPDH genes in human, mouse, and rat to estimate their rates of transitions and transversions in each species. We also applied this model on the pairs of syntenic pseudogenes between primates and rodents to estimate the rates of transitions and transversions in the GAPDH pseudogenes of each species (Table 2). Then we aligned each GAPDH pseudogene to its parent gene in the same genome and calculated the nucleotide difference in terms of transitions and transversions. By estimating nucleotide substitution rates for the GAPDH genes, our calculations compensated for mutations occurring after they diverged from a common ancestral gene and the ages of the pseudogenes were adjusted accordingly. From the nucleotide differences and the above estimated rates of transitions and transversions in genes and pseudogenes, we estimated the ages of the non-syntenic GAPDH pseudogenes, as shown in Figure 5. The ages of the non-spermatogenic GAPDH pseudogenes were not included, as they appeared to have become more severely degraded. These dating calculations are particularly sensitive to the quality of the underlying genome sequence and annotation. Consequently, we only report data for the three most completely finished and annotated genomes in our set: human, mouse, and rat. Because the chimpanzee genome diverged from the human genome so recently, we would not expect chimpanzee to have very different numbers for the comparison.

## Discussion

As a central pathway in metabolism, glycolysis has been highly conserved across multiple species from archaea to humans. The omnipresence of the glycolytic enzymes makes for a crude but standardized genomic measuring stick, comprising an ideal platform for studying pseudogenes.

Despite the high degree of conservation in the glycolytic enzymes, there is much more variation in their pseudogene abundances. Some genomes, like chicken, zebrafish, pufferfish, fruitfly, and worm, have very few or none, while others, like mouse and rat, have hundreds. The differences in pseudogene abundances alone suggests significant differences in the processes of gene expression, duplication, and retrotransposition in the different genomes. Previous studies have suggested that the difference lies in the prolonged lampbrush stage of oogenesis in mammalians as compared to non-mammalian organisms [48,49].

**Figure 6:**
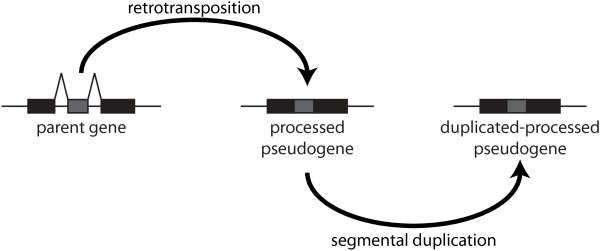
**Aetiology of a duplicated-processed pseudogene**. Alternative aetiology of a processed pseudogene. A parent gene is first retrotransposed into a processed pseudogene. Then the processed pseudogene undergoes segmental duplication to produce a duplicated-processed pseudogene.

Most glycolytic pseudogenes are processed and can be assumed to be retrotransposed from an mRNA intermediate. It is possible that certain sequences intrinsic to the GAPDH and LDH genes may predispose them to be preferentially retrotranscribed, inserted, and preserved in the genome. These pseudogenes are classified as processed and not duplicated indicating their formation was the result of a retrotransposition event of the parent gene, rather than a duplication event. However, we must consider the possibility of formation of a processed pseudogene through a retrotransposition event and its subsequent duplication giving rise to so called "duplicated-processed" pseudogenes. Thus, while duplicated pseudogenes result from the duplication of parent gene, duplicated-processed pseudogenes result from the duplication of a processed pseudogene [50,51]. One way to differentiate processed pseudogenes from duplicated-processed pseudogenes is to check if the segments of the genome surrounding a pair of processed pseudogenes are also similar. Hence, we checked for the presence of 60 processed pseudogenes of human GAPDH in duplicated regions of the genome called segmental duplications [52]. A pair of processed pseudogenes located in segmental duplication pairs indicates that one of the pseudogenes was likely formed by the duplication of the other one and hence is a duplicated-processed pseudogene (Figure 6). We identified eight duplicated-processed pseudogenes by this analysis, listed in Additional File 1. However, six of those eight pseudogenes occupy > 77% of the segments that are duplicated and could be the result of independent retrotransposition events. In this scenario perhaps the high sequence similarity of these segments led to their annotation as segmental duplications.

As a coincident finding, GAPDH has many more biological roles outside glycolysis as compared to the other glycolytic enzymes. For example, GAPDH functions in DNA repair, telomeric DNA binding, transcriptional regulation, nuclear RNA export, apoptosis, membrane fusion, phosphorylation, tubulin bundling, and sperm motility [53-59]. Because the molecular processes of retrotransposition are separate from the enzymatic functionalities, we can only speculate that the preponderance of non-glycolytic roles may be correlated to the enrichment of GAPDH pseudogenes.

In an intergenomic analysis, GAPDH pseudogenes have about five- to six-fold greater abundance in the rodent genomes as in the primate genomes even though overall the mouse genome was found to have about half as many pseudogenes as the human genome [3]. The mouse genome has higher rates of nucleotide substitution, insertion, and deletion [33] than the human genome, leading to a higher rate of pseudogene decay. However, the higher rate of pseudogene decay seems to have preferentially spared the GAPDH pseudogenes.

To further characterize the molecular history of pseudogenes in the human, chimpanzee, mouse, and rat genomes, it was necessary to identify the pseudogenes that were most likely present prior to the primate-rodent ancestral divergence. We used orthologous genes to identify regions of synteny between primate-rodent genome pairs. This approach is based on the assumption that gene-coding regions are much less variable than intergenic regions because of functional constraints and are therefore more reliably matched between genome pairs.

The scarcity of GAPDH pseudogenes syntenic between the primate and rodent genomes suggests an increase in retrotranspositional activity after the primate-rodent divergence 91 million years ago, which is consistent with the findings of previous investigators [6]. In order to achieve more detail in the timeline and provide further corroboration, we used Kimura's two-parameter model of nucleotide substitution to estimate the rates of change in the GAPDH genes and pseudogenes and thereby calculate the insertion date of each pseudogene. The creation dates formed three distinct distributions centered at 42.0, 36.3, and 25.9 million years ago in the human, mouse, and rat genomes, respectively, signifying a burst in retrotranspositional activity around those times. Kimura's model assumes neutrally evolving sequences, as in many pseudogenes [42], but some may initially be subject to natural selection [12] and the ages of these pseudogenes may be underestimated. In the human genome, the bursts in retrotranspositional activity may coincide with the "Alu burst" that occurred about 40 million years ago in primate genomes [60,1,5,61]. By examining the sensitivity of our pseudogene pipeline, as decribed under Methods, we found that the number of pseudogenes does not vary significantly with the threshold for sequence identity or BLAST score when compared to the parent gene. Thus, we believe this dating method accurately reflects all GAPDH pseudogenes and is not significantly biased towards longer and therefore younger pseudogenes.

## Conclusion

The ubiquitous nature of glycolytic enzymes rendered their pseudogenes most appropriate for comparing retrotransposition among multiple genomes. There was no evidence for preferential distribution of GAPDH pseudogenes in relation to individual chromosomes and to the location of the parent genes. We were able to calculate synteny using orthologous genes as anchors between two genomes. Whereas retrotransposition and gene annotation have been previously characterized on an individual genome basis, our syntenic method allowed us to perform a careful analysis of one pseudogene family across multiple genomes. This and a molecular clock analysis indicated that three distinct bursts in the insertion of GAPDH pseudogenes occurred at approximately 42, 36, and 26 million years ago in the human, mouse, and rat genomes, respectively, with evidence that most were created within the last 50 million years, subsequent to the divergence of rodent and primate lineages.

## Authors' contributions

YJL carried out the tabulation of processed and duplicated pseudogenes of glycolytic enzymes, syntenic and evolutionary analysis, and calculation of pseudogene ages. DZ, SB, NC, RR, and MBG were involved in developing and calibrating our pseudogene pipeline. EK carried out the analysis of potential duplicated processed pseudogenes in sequence-duplicated regions of the human genome. MBG conceived of the study and participated in its design and coordination. All authors read and approved the final manuscript.

## Supplementary Material

Additional file 1 **Supplement**. The sensitivity of our pseudogene pipeline is clarified and the sets of duplicated-processed pseudogenes are cataloged. Click here for file
