# Supplementary material for: Comprehensive analysis of the pseudogenes of glycolytic enzymes in vertebrates: the anomalously high number of GAPDH pseudogenes highlights a recent burst of retrotrans-positional activity
Source: BMC Genomics. 2009 Oct 16;10:480. doi: 10.1186/1471-2164-10-480 (PMC2770531; doi:10.1186/1471-2164-10-480)
Supplement: Additional file 1 — Supplement. The sensitivity of our pseudogene pipeline is clarified and the sets of duplicated-processed pseudogenes are cataloged. [file 1471-2164-10-480-S1.PDF]

Supplement

Pseudogene pipeline

We further analyzed the sensitivity of our pseudogene pipeline by varying the e-value threshold used to assign a potential BLAST pseudogenic match to a parent protein. There was a significant drop in matches as the e-value was lowered, as expected. At an e-value threshold 10^{-10}, 16,730 mouse pseudogenes were identified. When the threshold was raised to e-value of 1, i.e. when this parameter was ignored, the total number of pseudogenes increased only to 19,730. We chose to use the e-value threshold of 10^{-10} as it captured most of the matches.

Duplicated-processed pseudogenes

GAPDH processed pseudogenes were found in segmental duplications (SDs). Of eleven total regions, eight appear to have been segmentally duplicated from three original processed pseudogenes. Below we list three sets with each set likely containing one original processed pseudogene. However, since the segmental duplication data does not provide the direction of duplication, the original processed pseudogene within each set that likely gave rise to other duplicated-processed pseudogenes within the set cannot be distinguished.

1. G3P2_HUMAN_chr1_119813423, G3P2_HUMAN_chr1_119850835 (occupy 3–4% of the SDs)
2. G3P2_HUMAN_chr6_57795068, G3P2_HUMAN_chr6_58401927 (occupy ∼0.5% of the SDs)
3. G3P2_HUMAN_chr5_173872917, G3P2_HUMAN_chr23_39402613, G3P2_HUMAN_chr6_84159282, G3P2_HUMAN_chr6_80719791, G3P2_HUMAN_chr6_166448255, G3P2_HUMAN_chr23_46055398, G3P2_HUMAN_chr15_62607922 (occupy >77% of the SDs)
